# Supplementary material for: Content aware image restoration improves spatiotemporal resolution in luminescence imaging
Source: Commun Biol. 2023 May 13;6:518. doi: 10.1038/s42003-023-04886-z (PMC10183019; doi:10.1038/s42003-023-04886-z)
Supplement: Supplementary file 2 — Supplementary Information [file 42003_2023_4886_MOESM2_ESM.pdf]

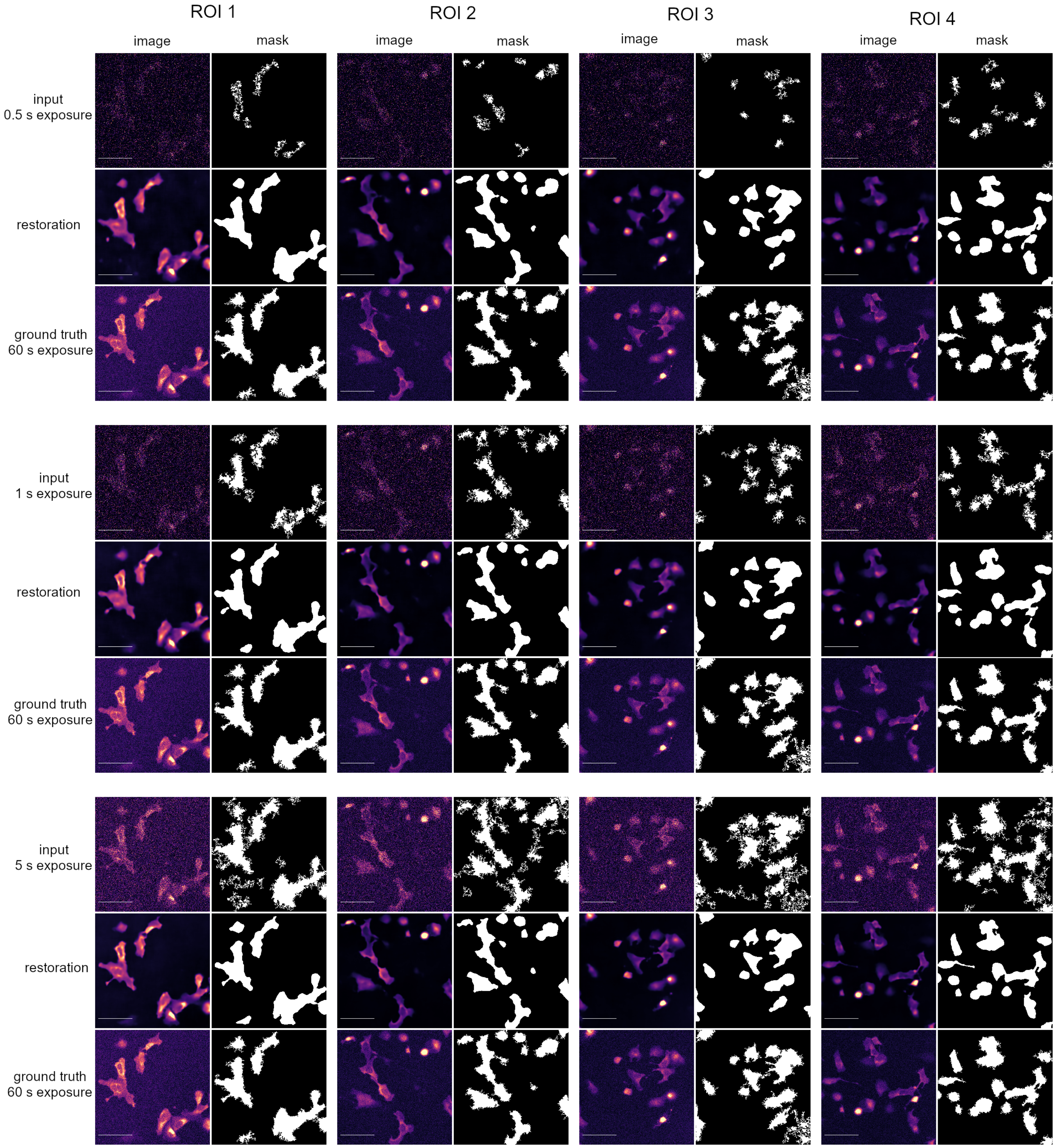

Supplementary figure 1 - Classical denoising for luminescence image restoration.
Content aware image restoration (CARE) allows the restoration of images obtained with short exposure times as indicated to images with a signal to noise ratio that is normally achieved with much longer exposure times. Restorations were performed on previously unseen data. Binary images represent automatically segmented objects from the respective micrograph. Scale bars = 10 µm

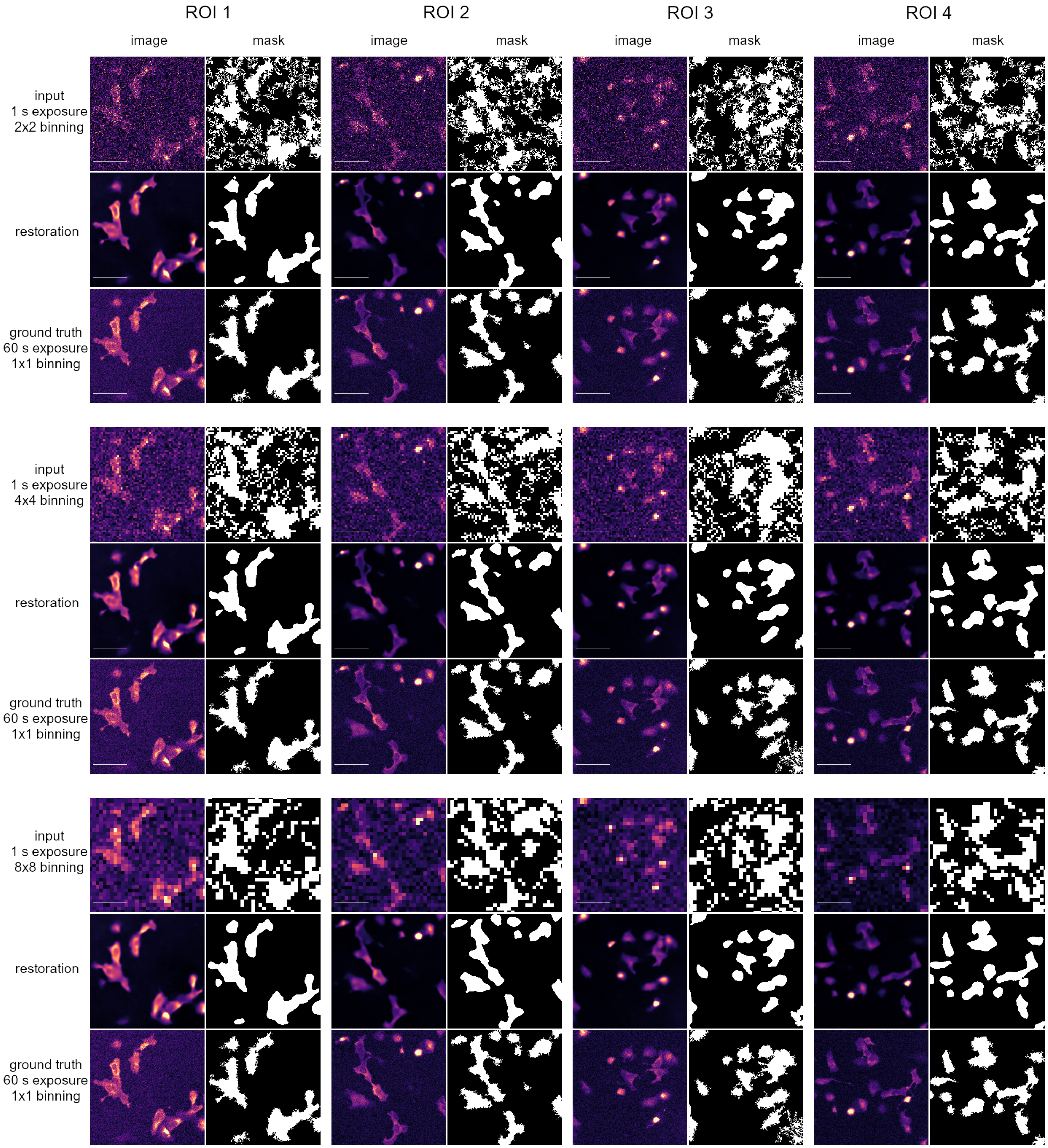

Supplementary figure 2 - Upsampling in luminescence image restoration.
CARE can be used to upsample and restore binned images to full resolution. Restorations were performed on previously unseen data. Binary images represent automatically segmented objects from the respective micrograph. Scale bars = 10 µm

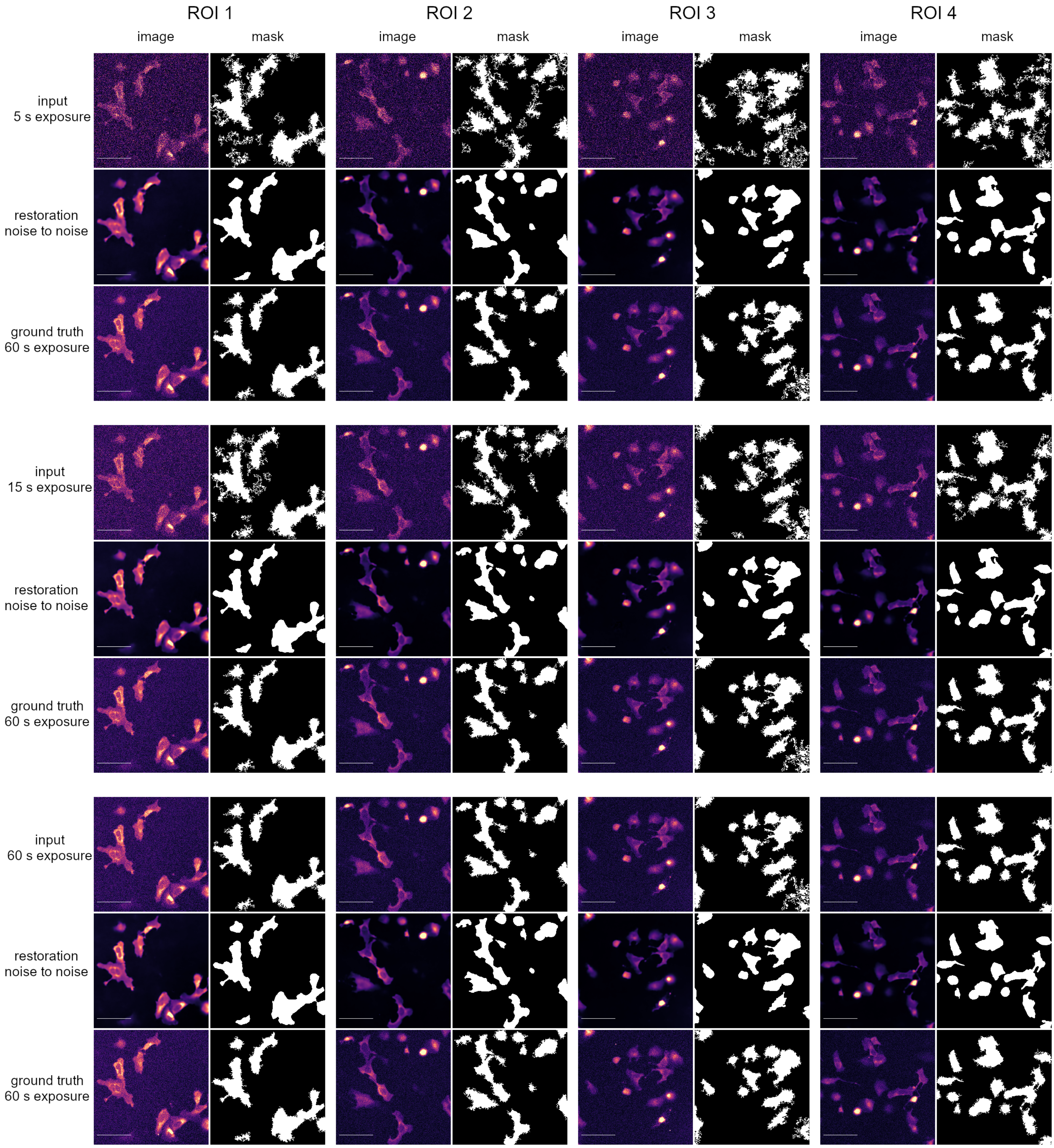

Supplementary figure 3 - noise2noise denoising in luminescence image restoration.
CARE can be utilised for noise2noise image restorations by training a network with image pairs of short exposure times only. Restorations were performed on previously unseen data. Binary images represent automatically segmented objects from the respective micrograph. Scale bars = 10 µm

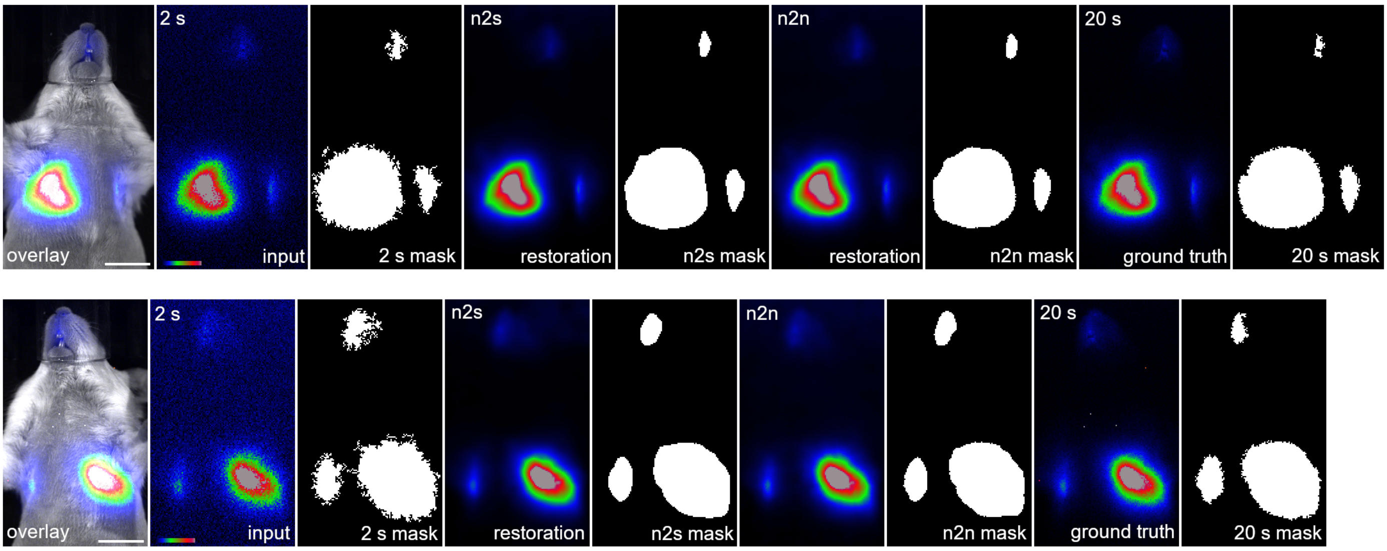

Supplementary figure 4 - Luminescence signal restoration in vivo.
Firefly luciferase signals can be restored from as few as 2 s to a signal to noise level corresponding to 20 s exposure time. noise2signal and noise2noise models were trained and applied. Scale bars = 1 cm

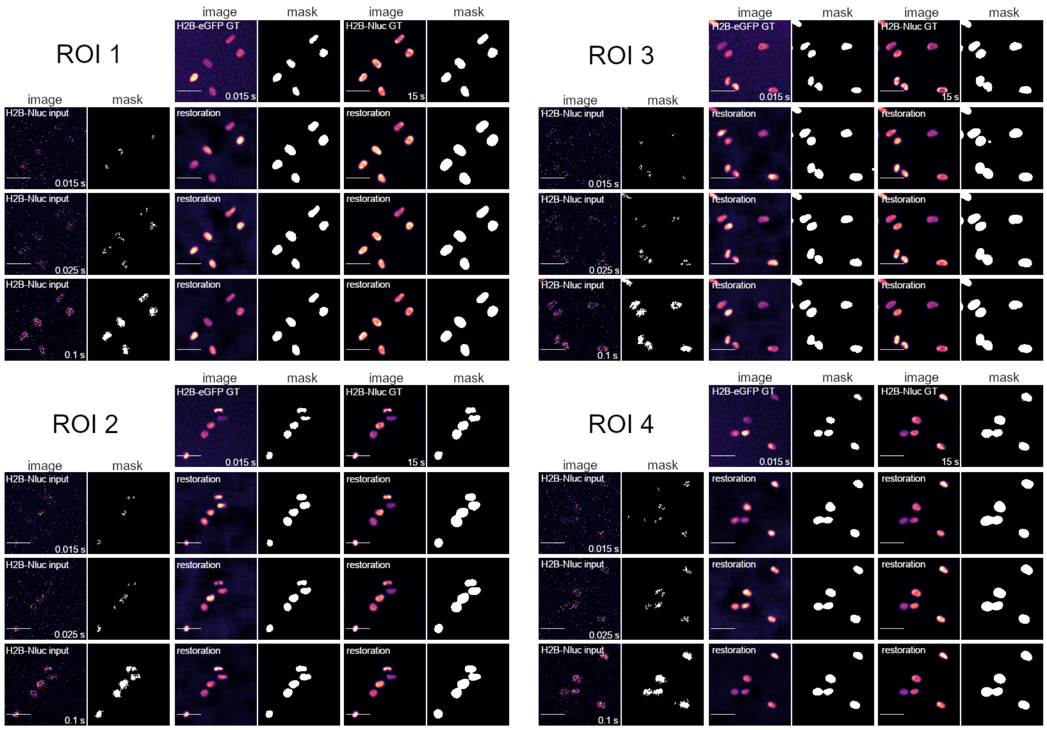

Supplementary figure 5 - Restoration of luminescence signals to fluorescent ground truth. Fluorescently labelled proteins can act as ground truth to reduce exposure times required for training data acquisition. H2B-Nluc and H2B-eGFP were cotransfected and training pairs were generated by capturing short exposure luminescence and short exposure fluorescence signals. Long exposure luminescence signals were recorded for quality control. Restorations were performed on previously unseen data. Binary images represent automatically segmented objects from the respective micrograph. Scale bars = 10 µm

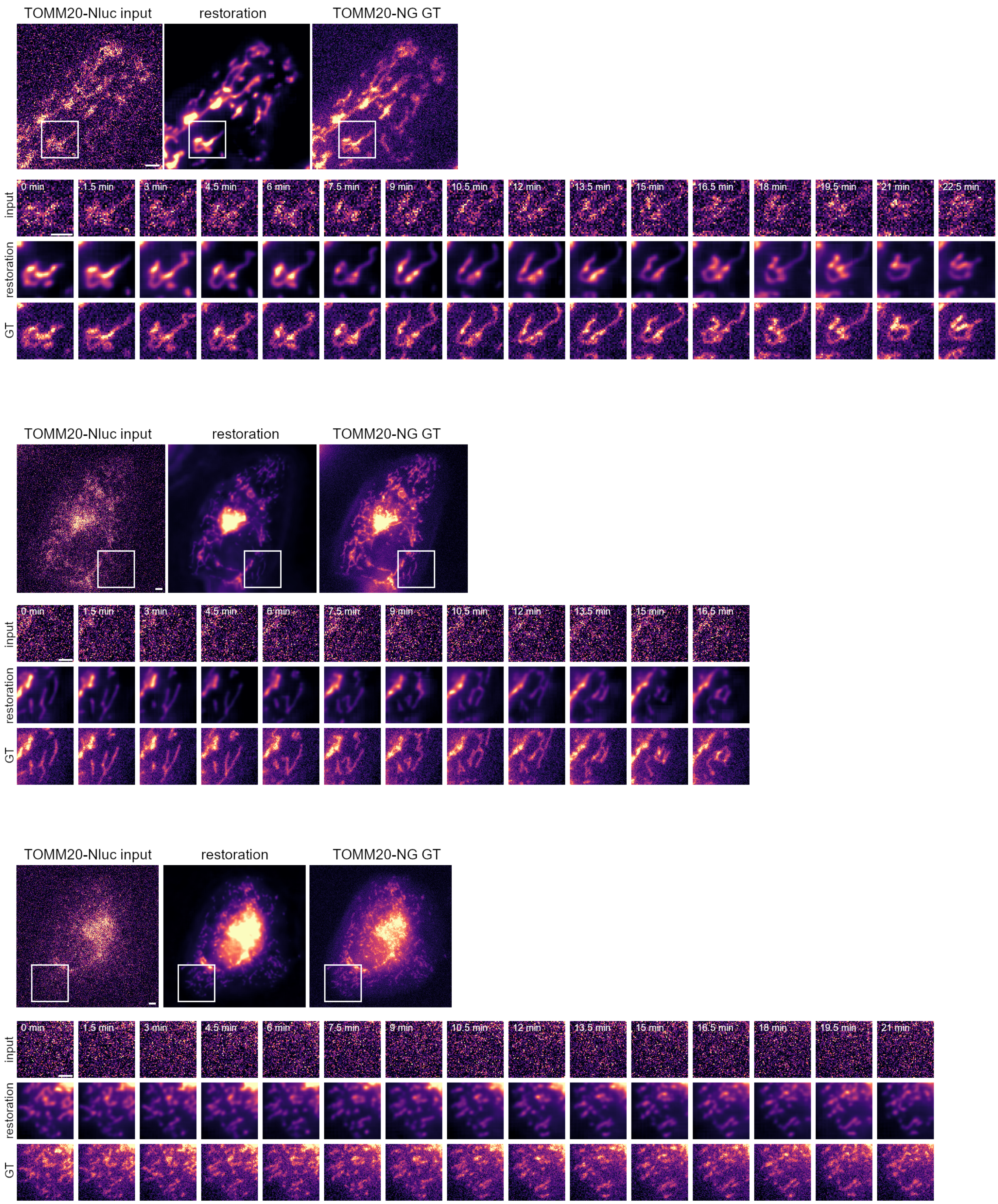

Supplementary figure 6 - Restoration of mitochondrial dynamics.
Examples of restored time lapse recordings of mitochondria labelled with TOMM20-Nluc (2 s exposure time). Shown are single optical slices. The network was trained with 3 dimensional stacks. The fluorescently labelled TOMM20-NG acts in training and validation as ground truth. Scale bars = 2 µm

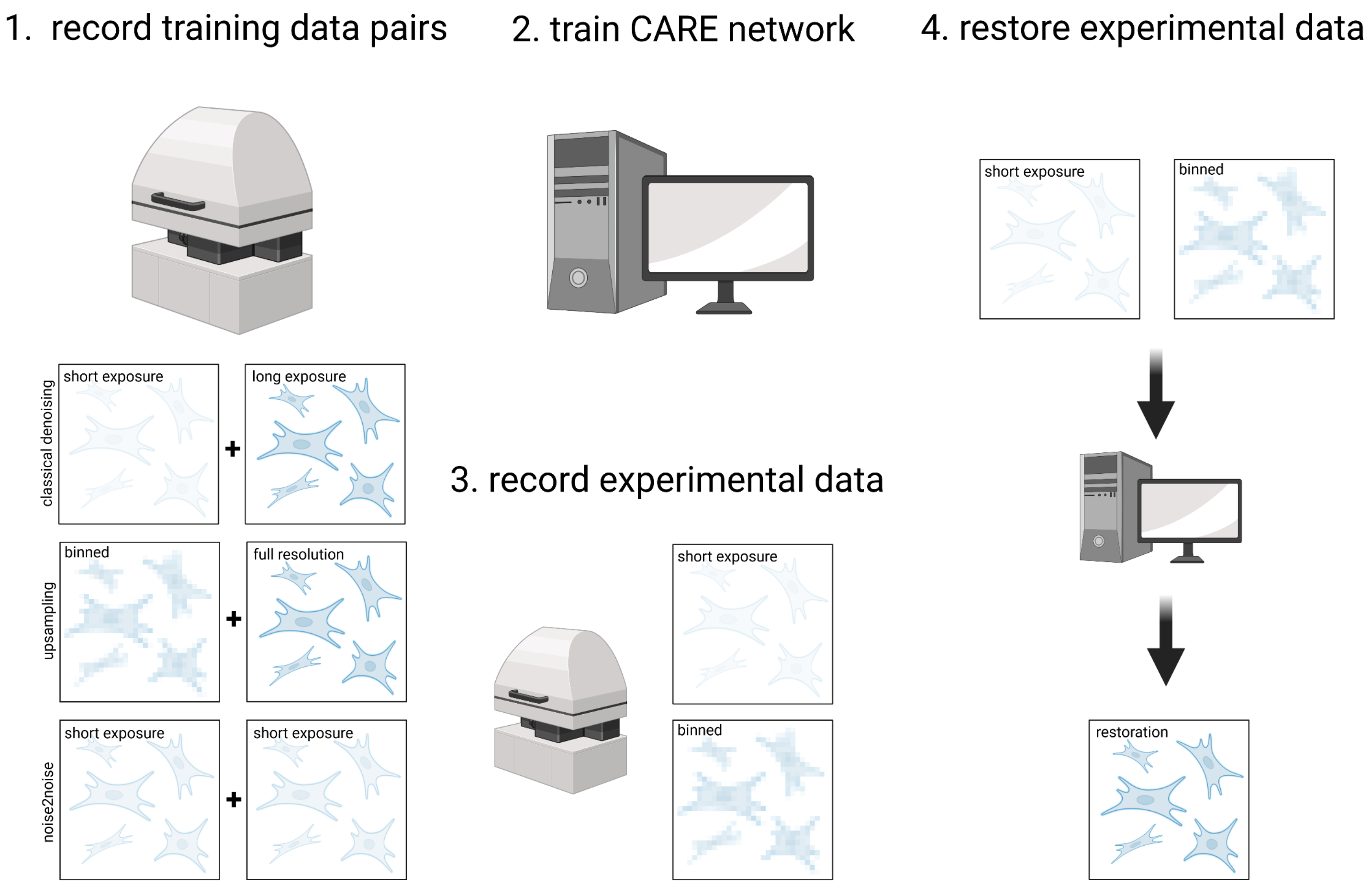

Supplementary figure 7 - CARE workflow.
Illustration of a standard CARE workflow for image restoration.

		Structures to restore			
		**Cytoplasmic Nluc**	**H2B-Nluc**	**Tomm20-Nluc**	***in vivo* Fluc**
Microscope parameters for generating raw training 100data	XYZ camera ROI (pixels)	1024x1024x16 (unbinned)	512x512x16 (unbinned)	512x512x16 (unbinned)	240x240x1 (8x8 binned)
	Z stepping	570 nm	570 nm	130 nm	NA
	Number of Stacks	8	14	90	22
	Objective	20x XAPO	20x XAPO	100x APO	NA
Processed Training data	Patch dimensions XYZ (pixels) (`patch_size`)	64x64x16	64x64x16	64x64x16	64x64x1
	number of randomly sampled patches per raw image stack (`n_patches_per_image`)	200	250	150	30
Training parameters	Number of epochs (`train_epochs`)	150	100	200	100
	Steps per epoch (`train_steps_per_epoch`)	200	400	600	100
	Batch size (`train_batch_size`)	128	128	128	128
	Fraction used for validation (`validation_split`)	0.1	0.2	0.3	0.1

Supplementary Table 1: Data acquisition and network training parameters.
