# Supplementary material for: Content aware image restoration improves spatiotemporal resolution in luminescence imaging
Source: Commun Biol. 2023 May 13;6:518. doi: 10.1038/s42003-023-04886-z (PMC10183019; doi:10.1038/s42003-023-04886-z)
Supplement: Supplementary file 3 — Description of Additional Supplementary Files [file 42003_2023_4886_MOESM3_ESM.pdf]

Description of Additional Supplementary Files

File name: Supplementary Data 1

Description: The numerical source data behind all graphs in the paper.
