# Supplementary material for: Content aware image restoration improves spatiotemporal resolution in luminescence imaging
Source: Commun Biol. 2023 May 13;6:518. doi: 10.1038/s42003-023-04886-z (PMC10183019; doi:10.1038/s42003-023-04886-z)
Supplement: Supplementary file 5 — Reporting Summary [file 42003_2023_4886_MOESM5_ESM.pdf]

nature portfolio

Corresponding author(s): Tobias Boothe
Jochen C Rink

Last updated by author(s): Apr 5, 2023

Reporting Summary

Nature Portfolio wishes to improve the reproducibility of the work that we publish. This form provides structure for consistency and transparency in reporting. For further information on Nature Portfolio policies, see our Editorial Policies and the Editorial Policy Checklist.

Statistics

For all statistical analyses, confirm that the following items are present in the figure legend, table legend, main text, or Methods section.

n/a	Confirmed	
☐	☒	The exact sample size (*n*) for each experimental group/condition, given as a discrete number and unit of measurement
☐	☒	A statement on whether measurements were taken from distinct samples or whether the same sample was measured repeatedly
☐	☒	The statistical test(s) used AND whether they are one- or two-sided<br>*Only common tests should be described solely by name; describe more complex techniques in the Methods section.*
☒	☐	A description of all covariates tested
☒	☐	A description of any assumptions or corrections, such as tests of normality and adjustment for multiple comparisons
☒	☐	A full description of the statistical parameters including central tendency (e.g. means) or other basic estimates (e.g. regression coefficient) AND variation (e.g. standard deviation) or associated estimates of uncertainty (e.g. confidence intervals)
☒	☐	For null hypothesis testing, the test statistic (e.g. *F*, *t*, *r*) with confidence intervals, effect sizes, degrees of freedom and *P* value noted<br>*Give P values as exact values whenever suitable.*
☒	☐	For Bayesian analysis, information on the choice of priors and Markov chain Monte Carlo settings
☒	☐	For hierarchical and complex designs, identification of the appropriate level for tests and full reporting of outcomes
☒	☐	Estimates of effect sizes (e.g. Cohen's *d*, Pearson's *r*), indicating how they were calculated

Our web collection on statistics for biologists contains articles on many of the points above.

Software and code

Policy information about availability of computer code

Data collection	The software used including their version numbers are stated in the method section of the manuscript
Data analysis	The software used including their version numbers are stated in the method section of the manuscript

For manuscripts utilizing custom algorithms or software that are central to the research but not yet described in published literature, software must be made available to editors and reviewers. We strongly encourage code deposition in a community repository (e.g. GitHub). See the Nature Portfolio guidelines for submitting code & software for further information.

Data

Policy information about availability of data

All manuscripts must include a data availability statement. This statement should provide the following information, where applicable:

- Accession codes, unique identifiers, or web links for publicly available datasets
- A description of any restrictions on data availability
- For clinical datasets or third party data, please ensure that the statement adheres to our policy

All raw data are available upon request to the corresponding authors. All source code used in this study is available at https://gitlab.gwdg.de/rinklab_public/lumicare.

Human research participants

Policy information about studies involving human research participants and Sex and Gender in Research.

Reporting on sex and gender	No human research participants in this study.
Population characteristics	No human research participants in this study.
Recruitment	No human research participants in this study.
Ethics oversight	No human research participants in this study.

Note that full information on the approval of the study protocol must also be provided in the manuscript.

Field-specific reporting

Please select the one below that is the best fit for your research. If you are not sure, read the appropriate sections before making your selection.

☒ Life sciences ☐ Behavioural & social sciences ☐ Ecological, evolutionary & environmental sciences

For a reference copy of the document with all sections, see nature.com/documents/nr-reporting-summary-flat.pdf

Life sciences study design

All studies must disclose on these points even when the disclosure is negative.

Sample size	For each of the experiments we chose the sample size such that training and test images are representative of the variability seen across all developmental time-points
Data exclusions	No data was excluded from the manuscript.
Replication	For all experiments we provide the code. Raw training data will be provided on request.
Randomization	For each experiment, the held-out test set was randomly selected from the corpus of data.
Blinding	For each experiment, the restoration models were optimized based on a random validation set that had no overlap with the held-out test set on which the final evaluation results were based.

Reporting for specific materials, systems and methods

We require information from authors about some types of materials, experimental systems and methods used in many studies. Here, indicate whether each material, system or method listed is relevant to your study. If you are not sure if a list item applies to your research, read the appropriate section before selecting a response.

Materials & experimental systems

n/a	Involved in the study	
☒	☐	Antibodies
☐	☒	Eukaryotic cell lines
☒	☐	Palaeontology and archaeology
☐	☒	Animals and other organisms
☒	☐	Clinical data
☒	☐	Dual use research of concern

Methods

n/a	Involved in the study	
☒	☐	ChIP-seq
☒	☐	Flow cytometry
☒	☐	MRI-based neuroimaging

Eukaryotic cell lines

Policy information about cell lines and Sex and Gender in Research

Cell line source(s)	U2OS and Hela CCL cells were a gift from Stefan Jakobs (Goettingen)
Authentication	None of the cell lines used were authenticated.

Mycoplasma contamination	All cell lines tested negative for mycoplasma contamination.
Commonly misidentified lines (See ICLAC register)	None of the cell lines used are listed as commonly misidentified.

Animals and other research organisms

Policy information about studies involving animals; ARRIVE guidelines recommended for reporting animal research, and Sex and Gender in Research

Laboratory animals	The mouse strain B6N-Tyrc-Brd/BrdCrCrl (Charles River) was used for this study
Wild animals	No wild animals were used in this study.
Reporting on sex	Sex was not considered in this study and is therefore not reported.
Field-collected samples	No field collected samples were used.
Ethics oversight	All animal in-vivo procedures were performed in compliance with the guidelines of the European Directive (2010/63/EU) and the German ethical laws and were approved by the administration of Lower Saxony, Germany (# G18/2773).

Note that full information on the approval of the study protocol must also be provided in the manuscript.
